# Dealing with complications in interventional radiology

**DOI:** 10.1186/s42155-024-00442-w

**Published:** 2024-03-21

**Authors:** A. O. Oseni, J.-Y. Chun, R. Morgan, L. Ratnam

**Affiliations:** 1 ST6 Interventional Radiology Fellow at St George’s Hospital NHS Trust, London, UK; 2 Consultant Diagnostic and Interventional Radiologist at St Georges Hospital NHS Trust, London, UK

## Abstract

It is widely accepted that most misadventures, which lead to harm have not occurred because of a single individual but rather due to a failure of process that results in healthcare workers making mistakes. This failure of process and the pervasiveness of adverse events is just as prevalent in Interventional Radiology (IR) as it is in other specialities. The true prevalence and prevailing aetiology of complications in IR are not exactly known as there is a paucity of investigative literature into this area; especially when compared with other more established disciplines such as Surgery. Some IR procedures have a higher risk profile than others. However, published data suggests that many adverse events in IR are preventable (55–84%) and frequently involve a device related complication such as improper usage or malfunction. This article aims to discuss factors that contribute to complications in IR along with tools and strategies for dealing with them to achieve optimal patient outcomes.

## Background

‘*To err is human’* was a landmark American publication released in 1999 which heralded the era of patient safety culture. It sought to overcome the stigma of medical errors by establishing a national agenda for improving patient safety through the implementation of a safer healthcare system [1]. Since then, several publications worldwide, including various systematic reviews and meta-analyses, have been published that reveal the prevalence of medical errors and adverse events in clinical practice [2–4]. According to the World Health Organisation, around one in every ten patients is harmed in healthcare; and more than three million deaths occur annually due to unsafe care. Approximately 50% of harm caused to patients is preventable [5–8]. Common sources of patient harm include medication; surgical and diagnostic errors; and healthcare associated infections. There are multiple factors that lead to patient harm including organisational, technological, human and patient related factors, as well as external factors (for example, the absence of robust policy and inconsistent regulation). It is widely accepted that most misadventures which lead to harm, have not occurred because of a single individual, but rather due to a failure of process that results in healthcare workers making mistakes.

This failure of process and the pervasiveness of adverse events is just as prevalent in Interventional Radiology (IR) as it is in other specialities. In 1964, when Dr Charles Dotter performed percutaneous angioplasty of a stenosed superficial femoral artery in an 82-year-old patient with gangrenous ischaemia, a new medical speciality was born [9]. There was a widespread transformation of formerly known diagnostic angiographers into interventional radiologists with rapid growth and expansion of the scope of practice over the past half century [10]. A plethora of minimally invasive techniques are now employed to treat increasingly complex pathology with excellent technical and clinical success rates. The true prevalence and prevailing aetiology of complications in IR are not exactly known as there is a paucity of investigative literature into this area; especially when compared with other more established disciplines such as Surgery [11–14]. Some IR procedures have a higher risk profile than others. However, published data suggests that many adverse events in IR are preventable (55–84%) and frequently involve a device related complication such as improper usage or malfunction [11]. Nevertheless, in general IR procedures are associated with low complication rates. This safety profile can be attributed partly to the leadership of national and international organizations, and to published standards of practice guidelines [15–20]. Widespread acceptance and knowledge of the content within these guidelines within the IR community represents a positive proactive shift toward embedding patient safety culture as a foundational tenet of IR practice [21]. However, beyond adherence to guidelines, global literature suggests that quality and service evaluation within IR is lacking [22]. In a survey of members of the Cardiovascular and Interventional Radiology Society of Europe (CIRSE), 47% of 150 respondents reported engagement in morbidity, mortality, and improvement meetings which we believe are crucial for dealing with complications, improving practice, and maintaining patient safety [20–23].

This article aims to discuss factors that contribute to complications in IR along with tools and strategies for dealing with them to achieve optimal patient outcomes.

## What is a complication in IR?

A complication in IR is defined as an *‘unintended harm that has resulted from an IR intervention which may or may not lead to an unintended change in clinical course.’* There is a subtle difference in its meaning when compared with another commonly used term ‘adverse event’. This term tends to include patient perception of an undesired event even if no clear harm has been done [18]. For the remainder of this article, we will use the term complication, with emphasis on events which cause an unintended change in clinical course. This change in clinical course may be a longer inpatient admission, a permanent life-changing disability or even death [19, 22]. There are several classification systems that combine clinical outcome with severity of sequelae, which provides an objective measure of the severity of the complication. Some complications occur due to error, and some occur despite best clinical practice [23].

The safety of patients who attend IR for a procedure is dependent on the prevalence, severity, and handling of complications in that local facility [1]. The rapid expansion of IR, the reliance on equipment and complex imaging means that there are several stages of intervention that are vulnerable to complications. Previous evaluation of complications in surgery have identified several contributory factors to the development of a complication that can be applied to IR practice. These are broadly divided into the following:

- Human factors- lack of technical competence, inadequate experience, inappropriate case selection, delay/failure to seek assistance, exhaustion/burnout [24, 25]
- System factors—poorly coordinated patient pathways, inadequate staffing levels, excessive caseload, poor communication, lack of robust on-call arrangements
- Equipment factors – Device malfunction, incorrect device usage, device usage outside the scope of instructions for use. In general, these equipment failures are thought to contribute to approximately 23% of intraoperative errors. Given the amount of equipment used in IR practice, we suggest that device failure contributes similarly or even more to the rate of complications seen in our discipline [26, 27]

The potential for complications increases in emergency procedures, where patients are haemodynamically unstable, and speed is of the essence. Emergency procedures frequently occur out of normal working hours, where there is less support from colleagues within IR and from other supportive disciplines. Patients presenting to IR for urgent procedures have often been deemed to be ‘unfit for surgery’, for example in cases of acute gastrointestinal haemorrhage or acute aortic syndromes. These patients are more likely to have significant co-morbidity further heightening the risk of intervention (Table 1).

**Table 1:** Categories of contributing factors in root cause analysis [21]

Category	Examples
**Human**	Primary operator inexperience Inappropriate case selection Imaging misinterpretation Deviation from instructions for use (IFU) or locally agreed protocols Failure of open disclosure Delayed recognition of clinical signs and symptoms
**Technical**	Challenging, technically difficult case Omission or error in the procedure
**System**	Unreliable access to IR suite Lack of robust on-call arrangements Poor access to inpatient beds Excessive caseload Limited access to anaesthetic support
**Education**	Poor Training Poor supervision of less qualified members of staff
**Patient**	Comorbidities ASA grade Non-compliance Refusal to consent to treatment
**Device**	Device malfunction Deviation from IFU (incorrect usage, or use outside the scope of IFU)
**Medication**	Side effect Error in drug administration

Surprisingly and counterintuitively, there can also be a significant risk of complication in procedures which are routinely practiced or those which are not felt to be technically challenging. Straightforward interventions can, and often do, cause the greatest harm to patients. Percutaneous arterial access is a basic skill in IR. However, reports suggest that up to 6% of patients experience complications following this intervention [28]. These include haematoma, pseudoaneurysm, haemorrhage and arterio-venous fistula [28]. The risk of complications is increased by several factors such as the number of previous interventions, anticoagulation, sheath size and closure method [29]. Another procedural example is IVC filter insertion, which is a widely practiced and technically straightforward procedure. Reported rates of complications are as high as 40% for specific complications such as IVC perforation and device malposition/migration [30, 31]. Even seemingly benign procedures such as percutaneous drainage procedures carry significant rates of complication as high as 10% [32]. Data published from the MACAFI Trial, which assessed the practice of percutaneous cholecystostomy (PC) in patients with acute cholecystitis in the United Kingdom are of substantial concern. Thirty day readmission rates were as high as 42% and mortality rates post-PC ranged between 11–19% [33]. Systematic review and meta-analyses of PC also suggests a threefold higher risk of mortality from PC versus emergency laparoscopic surgery [34]. This supports the opinion that the risk of complications does not lie simply in the technical difficulty of the procedure but also in the decision regarding whether to perform the procedure at all. When complications occur, depending on their severity, more invasive efforts to manage the complication can expose the patient to a greater cumulative risk of harm. IRs often need to enlist the help of surgical colleagues to ‘fix’ a complication (e.g., arterial rupture, bowel perforation, visceral ischemia in the context of non-target embolization). With every additional intervention, the risk of further complication also increases. We are reminded of the words of the famous mathematician Augustus De Morgan (1866), popularly referred to as ‘Murphy’s Law’; “Anything that can go wrong, will go wrong”.

Aside from the effect of complications on patients, there are adverse psychological consequences of complications for the responsible physician. The practicing physician will often have to work through feelings of guilt and self-doubt that can have far-reaching emotional, cognitive, and physical ramifications culminating in a syndrome known as ‘Second Victim Syndrome’ [35, 36]. First coined by Dr Albert Au, this describes the burdens of anxiety, depression, and shame that any health care provider can feel after any traumatic adverse event [35, 36]. This feeling can influence our approach to dealing with complications with a hesitancy to recognize or even admit the medical error, for fear that it will expose us to malpractice liability [12, 37–45]. This occurs despite evidence that shows that patients appreciate honest disclosure of adverse events. Even in the United States where there is a pervasive culture of litigation, only 12% of complications lead to formal hearing [12, 38–42].

**Fig. 1:**
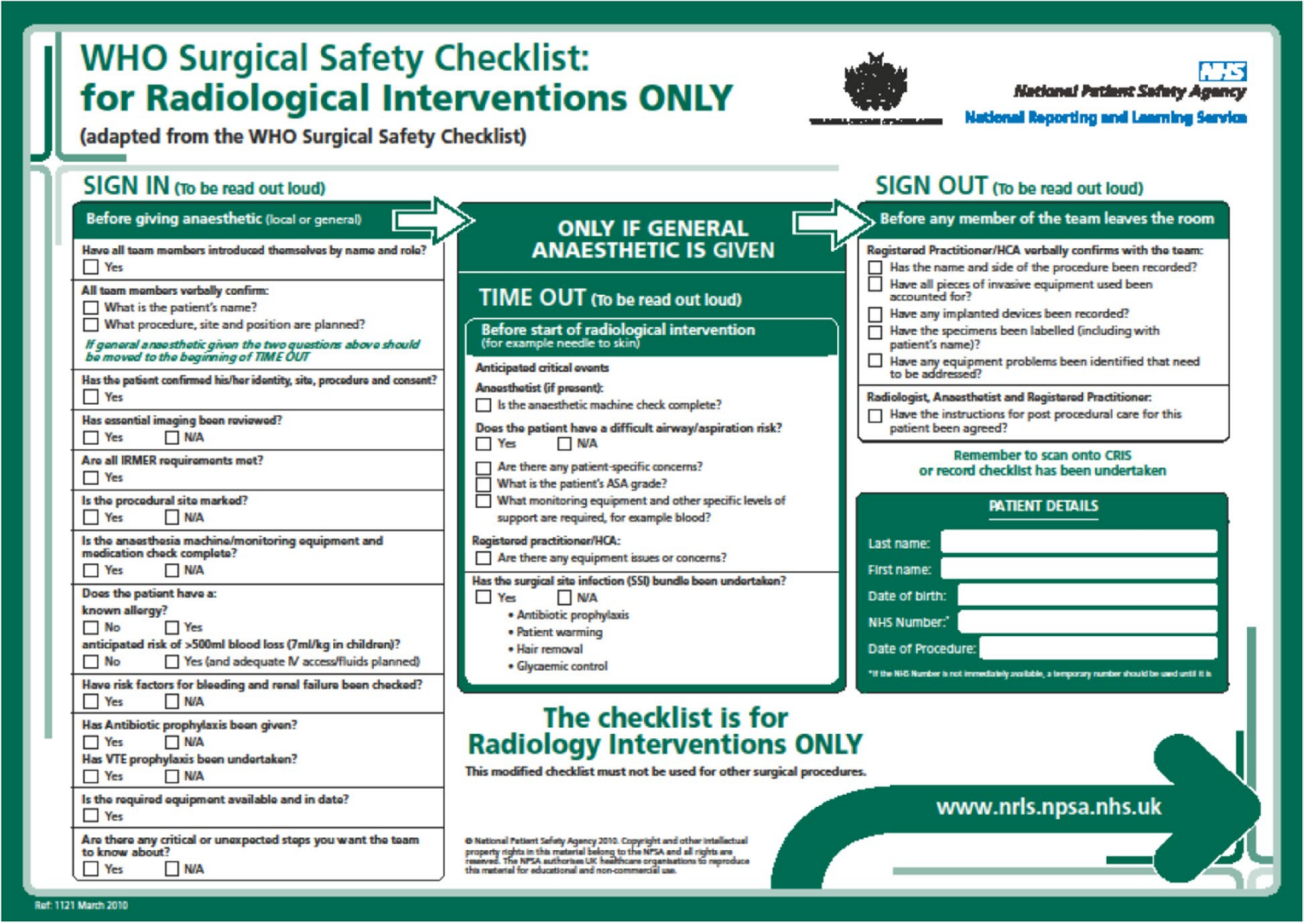
WHO Safety Checklist [46]

A proactive and pre-emptive approach to dealing with complications is crucial to safe practice. Robust pre-procedural discussion and planning as well as enlisting tools such as safety checklists significantly contributes to establishing a culture of patient safety in IR practice (Fig. 1: WHO checklist) [21, 45–47]. When complications have occurred, they should be reviewed and discussed at regular Morbidity and Mortality (M&M) meetings [21, 47]. These are an important part of the governance framework to ensure shared learning and to identify systems and processes that warrant change [21]. The remainder of this article will explore a robust framework for dealing with complications as they occur with the aim of helping IRs to manage complications in their day-to-day practice.

## A three-fold approach to dealing with complications in IR

### Recognising complications

The initial reaction to the occurrence of a complication is often shock or even denial [36, 39]. This is more likely if the complication is sudden or unexpected (never encountered before) and is influenced by the breadth of experience of the practitioner. Owning up to the mistake and asking for help can be difficult in the face of competing interests, such as pride and the need for self-preservation conflicting with shame and guilt at harm caused to the patient [40, 44].

Opportunities to recognize a complication can be divided into ‘intraprocedural’ and ‘post-procedural’. ‘Intraprocedural’ opportunities are linked with the methodology of the procedure, with key stages of operation serving as checkpoints for the advent of any error. The manifestation of the complication is often immediate and obvious. This lends itself to rapid intervention and timely correction of the error during the procedure which itself can minimize the wider ramifications of the complication. The IR responsible can often solve the complication independently, with the help of a second operator, or advice from other IRs in the department. More rarely, the help of other specialty colleagues such as vascular or general surgeons may be needed.

‘Post-procedural’ complications can be more challenging with most occurring within the first 24 h after the intervention, but many can occur days to weeks after the initial intervention. This calls for stringent post-procedural monitoring in the care of healthcare practitioners who understand the unique risks of the intervention that has been undertaken. Clear post-procedural protocols must exist especially in the context of ambulatory patient pathways, where patients may not be in a hospital when the complication occurs, for example:

- Post-procedure ward rounds are fundamental in maintaining the duty of care towards our patients and provide an invaluable opportunity to recognize any post-procedural complications.
- Day case patients should be contacted soon after intervention. In our institution, allied healthcare practitioners perform early follow-up telephone clinics for our ambulatory/day-case patients.
- Patients should be given procedure-specific information sheets with details of signs and symptoms to look out for which may herald a complication. Patients are also given contact details for our team in case of an emergency or any ongoing concerns.

Imaging is used in most IR procedures and is essential in the quest for better procedural outcomes. Final images which confirm device positioning, or the site of percutaneous vascular access serve as key problem-solving tools in the event of subsequent complications. Imaging can also be used when the expected clinical course takes a negative turn. For example, a patient in pain after a liver biopsy may need an urgent ultrasound to assess for complications such as subcapsular hematoma or biliary leak. A patient in pain after gastrostomy insertion may need a CT scan or Tubogram to assess tube positioning and safety for feeding.

The importance of good medical documentation is a fundamental tenet of good medical practice and is mandated by regulatory bodies [48]. The use of stored images to evidence interventions undertaken on our patients is a crucial part of this. As clinical notes are deemed to be legally binding documentation of a patient’s clinical course, completion images of our interventions are often the only other objective proof we have for evidencing, assuring, and at times defending our practice.

### Managing complications

Once a complication is recognized, the next step is to expediently manage the complication [49]. The ability to manage a complication depends on the experience of the practitioner (ranging from Consultants to IRs in training), the level of available support or supervision, and the robustness of pre-agreed in-house escalation protocols [46–50]. Varied approaches may be employed depending on the perceived risk of the complication at the time of its occurrence. A few case specific tips on how to manage complication have been include later in this manuscript.

#### Categories of decision making on ‘hot to manage a complication’ include

A. I have seen this before and the risk is of morbidity and mortality is low > conservatively manage and monitor for signs of deterioration.
B. I have not seen this before but based on experience the risk of subsequent morbidity is low > conservatively manage, monitor for signs of deterioration, consult experienced colleagues on best next steps.
C. I have seen this before; the risk of morbidity and mortality is high > execute a rescue plan of action (based on experience level of the practitioner) with or without the help of other experienced colleagues or other specialities.
D. I have not seen this before; the risk of morbidity & mortality is high, but the risk of further intervention is higher especially in absence of prior experience > more help should be enlisted either from other experienced IR colleagues or other specialities.

Adequate training and supervision are also crucial for equipping IRs with the necessary skill set to manage a complication when it occurs. Case based textbooks, IR journals, hands-on training courses, conferences and formal avenues of certification all serve to help expose practitioners to a breadth of case load which will help in the management of complications in day-to-day practice. [49]. The supplementary and voluntary EBIR examination administered by CIRSE is a good example of accreditation for IRs around the world, wherein the breadth and depth of IR practice is examined against rigorous standards and a curriculum which is linked to teaching materials made available by the society [50].

### Learning from a complication

After a complication has occurred, the next goal is to establish measures to prevent the recurrence of similar events. M&M meetings are an important part of the process of examining adverse outcomes with view to addressing any underlying issues. When conducted regularly and appropriately, M&M meetings been shown to promote shared learning and improve patient outcomes [21]. A root cause analysis is followed, a process of information gathering followed by a systematic identification of all contributing factors that led to the adverse event. Complications should be reviewed openly, where the practitioner is encouraged to reflect upon the case and seek comments and insights from other experienced colleagues in the department. These discussions are critical for shared learning and for development of institutional protocols. Conclusions are drawn by consensus regarding the type of error and seriousness of the complication based on the clinical outcome and severity of sequelae ([23], Table 2).

**Table 2:** Established frameworks for classification of complications

Grade	Description
**(a) CIRSE classification system for complications in IR**	
**1**	Complication during the procedure which could be solved within the same session; no additional therapy, no post-procedure sequelae, no deviation from the normal post therapeutic course
**2**	Prolonged observation including overnight stay (as a deviation from the normal post-therapeutic course < 48 h); no additional post procedure therapy, no post procedure sequelae
**3**	Additional post procedure therapy or prolonged hospital stay (> 48 h) required, no post procedure sequelae
**4**	Complication causing a permanent mild sequela (resuming work and independent living)
**5**	Complication causing a permanent severe sequela (requiring ongoing assistance in daily life)
**6**	Death
**(b) SIR classification of complications**	
**Minor Complications**	
**A**	No therapy, no consequence
**B**	Nominal therapy, no consequence; includes overnight admission for observation only
**Major Complications**	
**C**	Require therapy, brief hospitalization (< 48 h)
**D**	Major therapy, unplanned increased level of care, prolonged hospitalization (> 48 h)
**E**	Permanent sequelae
**F**	Death

**Fig. 2:**
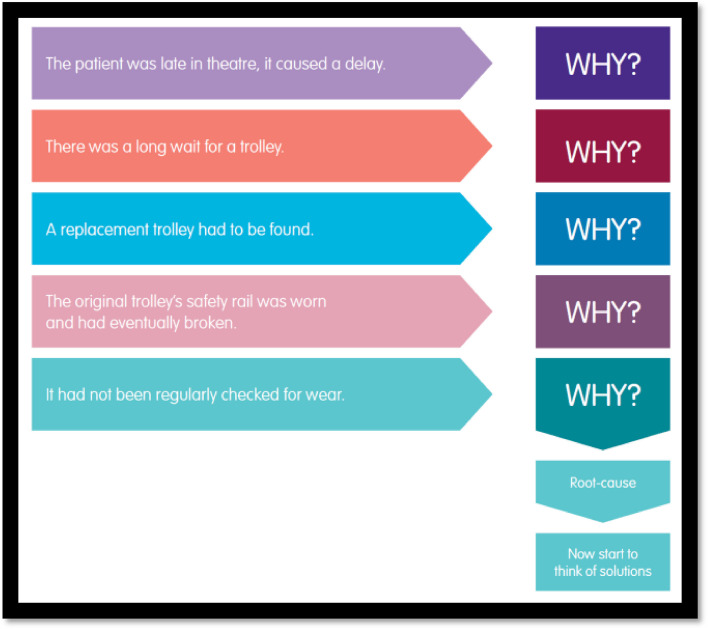
‘Five why’s- a practical example for how to determine the root cause of an adverse event. Source- The 'Five Whys' Analysis—Health Innovation West of England (healthinnowest.net)

Recommendations are then made about what can be done (if anything at all) to prevent the error from occurring again. These may include further education and training, raising awareness of recurring issues within the department, changing patient pathways and protocols, escalation to hospital-wide governance team or assisting with an ongoing investigation of a serious incident. Any adverse incidents related to medical devices should be reported to the manufacturer and the appropriate regulatory agency, such as the Medicines and Healthcare products Regulatory Agency (MHRA) in the United Kingdom (Fig. 2).

#### Duty of candour

‘Duty of candour’ is a regulation described in the Health and Social Care Act, UK 2008, which defines how healthcare practitioners should approach interactions with patients in the context of an adverse event. It stipulates that health care practitioners (registered persons) should be ‘open and transparent’ with patients (service users), making them aware of any unintended events that could have led to death, prolonged psychological harm, or severe/moderate physical harm [46, 48]. This is a legal requirement of practice in the UK and responsible physicians are tasked to own up to their mistakes and be honest with their patients when unintended events occur (Table 3).

**Table 3:** Key elements to consider when discussing adverse events with patients

**Content**	Relevant information is communicated concisely in a way that a patient and their family can understand and retain
**Timing**	The timing of information delivery is also important. For example, on the recovery unit after being roused from a general anaesthetic may not be the best time to inform patients about adverse intraprocedural events. Conversely such information should not be withheld for excessive periods of time, such as after a patient has been discharged from hospital
**Location**	The location in which information is shared should also be considered. Explaining the events leading to a relative’s death should be undertaken with great care and compassion, with preparations made for a quiet and private clinic room and adequate time to allow for any further questions or discussion
**Documentation**	Clear documentation of adverse events and relevant discussion should be included in the patients' clinical notes. Where relevant to ongoing follow up or intervention, the documentation should be included in the discharge paperwork
**Follow-up**	If a complication has led to death or a serious complication, a review meeting with the patient or relatives can be held to discuss results of any investigations into the case. This is often an effective way to provide closure for both the physician and the affected parties. These meetings often happen weeks to months after the initial event. Various thoughts or concerns can be explored in a constructive way after there has been time to reflect on events and conclude an investigation

**Fig. 3:**
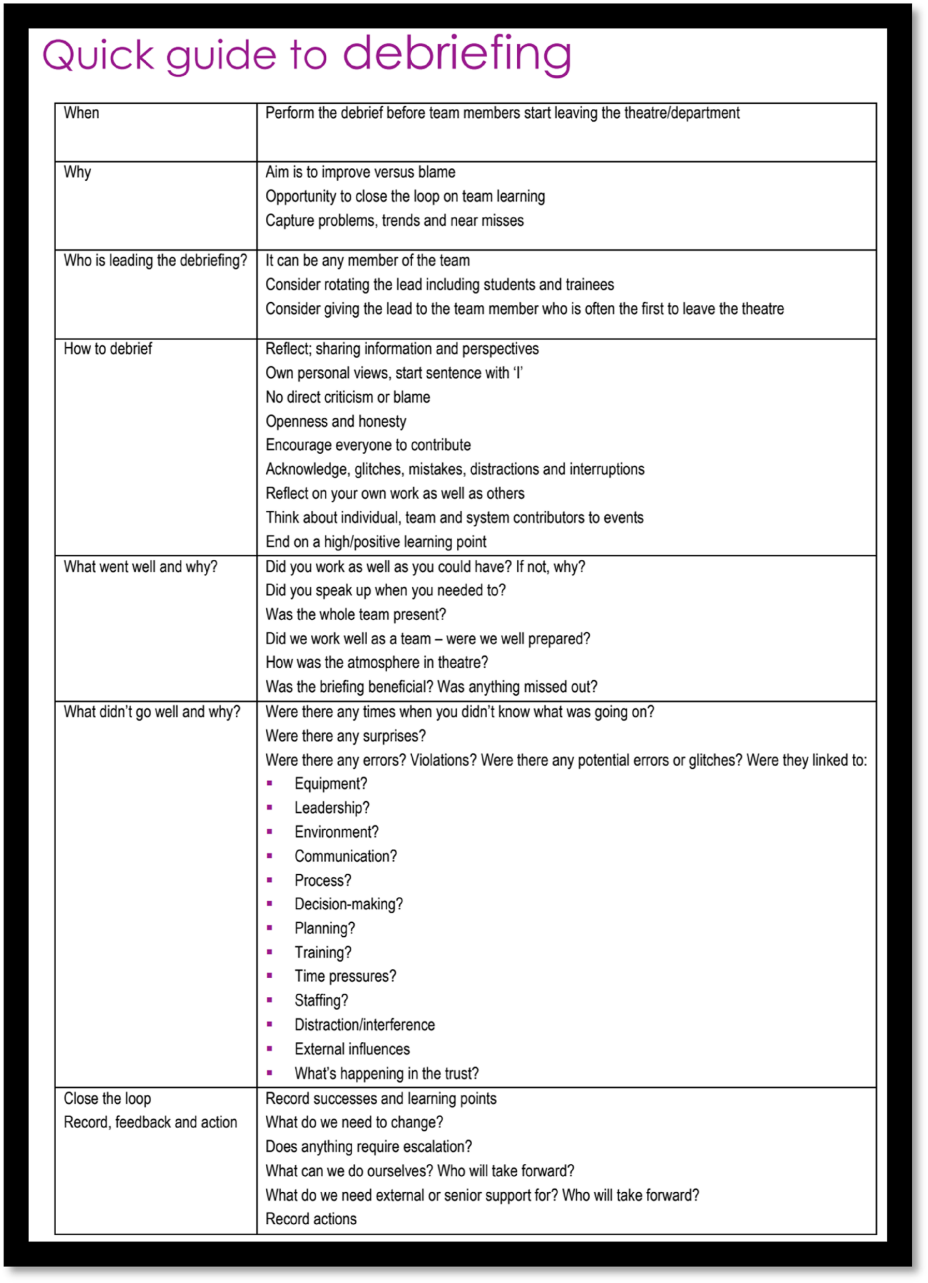
Suggestions for conducting a debrief [46, 48]

With the evolution and explosion of modern IR techniques it is difficult to provide an exhaustive examination of how do deal with all complications, however a few examples of complications encountered in our local institution have been detailed to serve as examples of what to look out for and what to do. For the purposes of this illustration, basic patient preparations such as pre-operative blood tests, antibiotic prophylaxis etc. are not fully detailed. Specifics for the type of intervention are discussed to illustrate points with regards to dealing with complications. This is not intended to be a comprehensive outline on performing the listed procedure (Fig. 3).

## Case 1

*Intervention:* Angioplasty/Stenting of an Iliac Artery Occlusion or Stenosis.

**Fig. 4:**
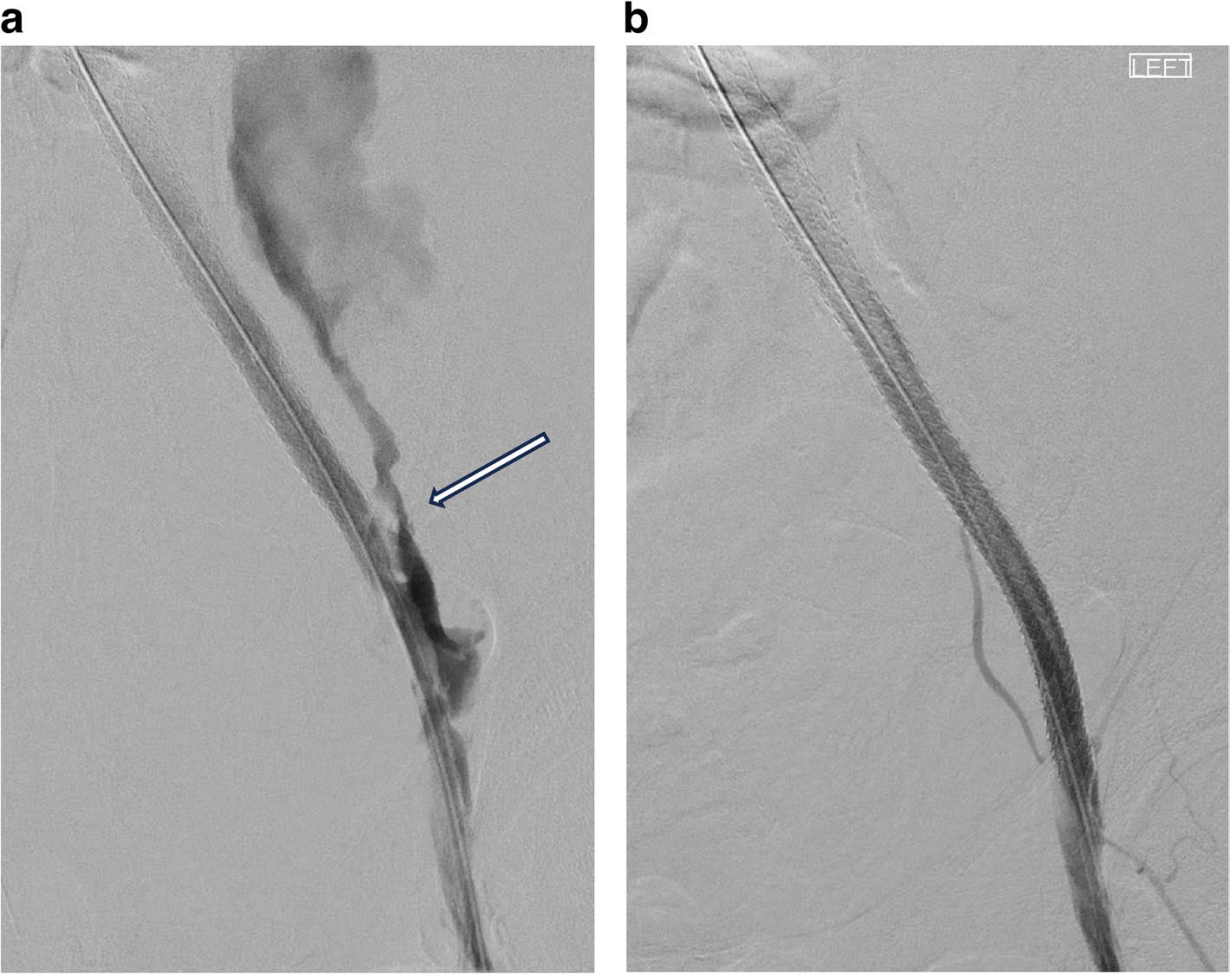
**a** External iliac artery rupture post angioplasty (shown by white arrow). **b** External iliac artery following deployment of a covered stent to seal the site of rupture

*Potential complications*: Iliac artery rupture, distal embolization, balloon-mounted stent dislodged from the balloon. These should always be anticipated when performing angioplasty or stenting for iliac arteries (Fig. 4).

### Pre- procedure planning

- Ensure appropriately sized covered stent and sheaths are available (this procedure should not be embarked on in an elective situation when this is not available)
- Obtain bilateral femoral arterial access.
  o gives better control of the situation should there be a rupture.
  o balloon occlusion and subsequent covered stent deployment can be conducted from ipsilateral access.
  o Imaging can be obtained from contralateral access.
  o Reduces the risk of balloon mounted stents becoming dislodged when introduced over the aortic bifurcation.
  o Ipsilateral sheath prevents distal embolization.

### Peri – procedure

- If an occlusion is crossed from the contralateral side, consider snaring wire so ipsilateral access is available for stent deployment.
- Following angioplasty of a stenosis or occlusion, persistent or worsening pain reported by the patient after deflation of the angioplasty balloon should alert the operator to the possibility of a rupture.
- Deflated balloon should be left on the wire just outside the sheath, while an angiogram is rapidly obtained.
- If a rupture is identified, the angioplasty balloon can be reinflated across the rupture or occlusion balloon inflated above rupture point while covered stent graft is prepared.
- Entire team should be made aware of the complication, resuscitation commenced, a second operator enlisted to assist if possible.
- Covered stent deployed across the rupture.
- Vascular surgical team made aware of situation in case surgical intervention is required.

### Post procedure

- Communicate with the clinical team, appropriate resuscitation and blood products administered as required.
- Documentation of procedure, complication and how it was dealt with.
- Discussion with patient and family where appropriate, explaining complication and what was done. Can be done briefly immediately after but should be done at a time after patient has recovered e.g., the next day on the ward.
- Team debriefs meeting: discussion of what went well and what could be improved on.
- Case review in mortality & morbidity meeting with shared learning points.

## Case 2

*Intervention*: Radiologically inserted gastrostomy (RIG)

**Fig. 5:**
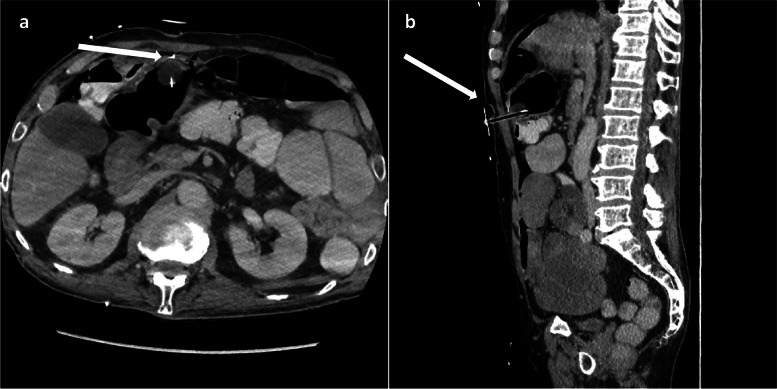
**a**, **b** Axial and sagittal CT images for patient who deteriorated on the ward post RIG insertion. White arrows show the gastrostomy tube traversing collapsed transverse colon

*Potential Complications*: Non target injury (colonic transection/ liver injury), inadequate fixation of gastrostomy balloon against abdominal wall leading to peritonism from leakage of gastric contents, complication of conscious intravenous sedation in patients with neurological conditions (Fig. 5).

### Pre- procedure planning

- Review previous imaging, ensure no anatomical abnormalities, anticipate difficulties e.g., hiatus hernia, previous gastric surgery, enlarged left lobe of liver.
- Ensure specific patient medical history is considered e.g., for patients with motor neurone disease, sedation should be used with utmost care due to the risk of respiratory depression – emphasis should be on good analgesia without the use of benzodiazepines.
- Ultrasound marking of liver edge before beginning the procedure.
- Administration of oral contrast pre-procedurally to outline the colon (timing must be correct for this to work).

### Peri – procedure

- Use of hyoscine butylbromide to reduce gastric emptying and reduce filling of small bowel with air.
- Screening gastropexy needle insertion in AP and lateral to ensure no bowel interposed between stomach and abdominal wall.
- Use of on table Dyna CT to ensure no transgression of bowel prior to serial dilatation of percutaneous tract.
- Ensure gastrostomy balloon is inflated with correct volume and pulled back against the anterior abdominal wall.

### Post procedure

**Fig. 6:**
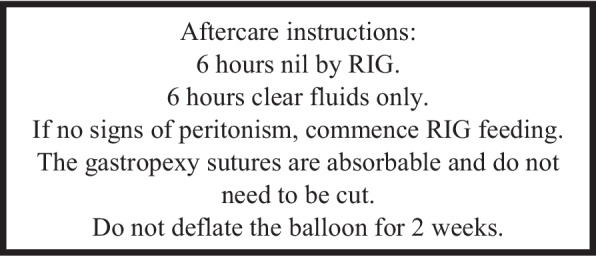
Sample of standard after care instructions documented post gastrostomy:

- Ensure written protocols are in place and post procedural instructions are clear with regards to safe feeding via the tube (Fig. 6)
- Ensure clear instructions regarding deflation of balloon and release of gastropexy sutures to ensure tract matures before this is done.
- If patient experiences significant pain or discomfort, have a low threshold for performing a CT or Tubogram (Fig. 6).
- Clinical teams must be made aware of the importance of contacting Interventional Radiology if there are any problems with the tube.

**Fig. 7:**
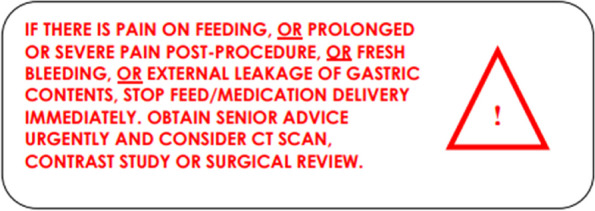
Recommended documentation labels for gastrotomy patients to warn of potential life-threatening complications [47]

Complications do occur, but if all involved in their care are vigilant and know when to act, this can avert serious morbidity and even mortality in this patient group (Fig. 7).

## Conclusion

Complications are an inevitability in clinical practice that require an open, conscientious, and systematic approach to their management. IRs should adopt a proactive approach to deal with complications by making sure that even before a procedure begins, every consideration is given to the things that may go wrong and how to deal with them. Planning meetings, MDTs and morning case reviews serve as helpful opportunities to consider the potential pitfalls of a case and what to do should these occur. IRs should be adequately trained and supervised or have the appropriate experience to recognize complications during procedures. We should operate with a clear framework of how-to assess a complication when it occurs, how to correct it and when to escalate to other people. With the shift to more day case procedures, robust pathways for managing post-procedure complications should be in place, with patients being adequately informed of symptoms to watch for, what to do and how to contact the department in case of emergency.

As IR continues to lead the frontiers of modern medicine, we need to be prepared to take greater ownership of our patients which includes dealing with and acknowledging our errors. We need to be humble enough to admit our limitations and be prepared to call for help whenever necessary. Humility also allows for an honest and open atmosphere of reflection and self-critique that is so crucial for establishing a good patient safety culture. When we are honest with ourselves, our colleagues and with our patients, we can aim for the highest standards of patient safety which will translate into a better patient experience and improved patient outcomes.

## Data Availability

Not applicable.

## Abbreviations

AP: Anterior posterior
CIRSE: Cardiovascular society if interventional radiology Europe
CT: Computed Tomography
EBIR: European Board of Interventional Radiology
IFU: Instructions for use
IR: Interventional Radiology
IVC: Inferior Vena Cava
MDT: Multi-Disciplinary Team
PC: Percutaneous Cholecystostomy
WHO: World Health Organization
